# Homologous Recombination Proficiency in High-Grade Serous Epithelial Ovarian Cancer Tumors: The Dark Side of the Moon

**DOI:** 10.3390/cimb47090702

**Published:** 2025-09-01

**Authors:** Marina Pavanello, Carolina Martins Vieira, Martina Parenza Arenhardt, Angelica Nogueira-Rodrigues

**Affiliations:** 1 School of Clinical Medicine, University of New South Wales, Sydney, NSW 2052, Australia; m.pavanello@unsw.edu.au; 2 Oncoclinicas, Belo Horizonte 30360-680, MG, Brazil; carolina.martins@medicos.oncoclinicas.com; 3 Irineu Boff Family Oncology Center, Nora Teixeira Hospital, Santa Casa de Porto Alegre, Porto Alegre 90035-074, RS, Brazil; tinaparenza@gmail.com; 4 Department of General Medicine, Federal University of Minas Gerais, Belo Horizonte 30130-100, MG, Brazil

**Keywords:** epithelial ovarian cancer, homologous recombination, molecular targeted therapies

## Abstract

Extensive research on homologous-recombination-deficient (HRD) tumors has led to advancements in targeted therapies, such as PARP inhibitors (PARPis). Around 50% of high-grade serous ovarian cancer (HGSOC) cases exhibit HR deficiency, but understanding the remaining half, referred to as homologous-recombination-proficient (HRP) tumors, is limited. This review explores existing knowledge regarding HGSOC patients with HRP tumors and offers insights into potential targets for innovative treatments. Patients with HRP tumors do not experience the same benefits from PARPi and have poorer survival outcomes compared to those with HRD tumors. *CCNE1* amplification is a common, well-established molecular feature in HGSOC HRP tumors, occurring in about 20% of cases. Targeting *CCNE1* amplification and/or overexpression shows promise with emerging therapies like CDK2 or Wee1 inhibitors. Additionally, approaches using immunotherapy and antibody–drug conjugates could represent promising targets for HRP patients. This review also covers lesser-known molecular features in HRP tumors, such as fold-back inversions and *CARM1* amplification and/or overexpression, as well as HRD tumors that acquire HR proficiency (*BRCA1/2* reversion mutations, demethylation of *BRCA1* and *RAD51C*). We also discuss controversial topics regarding HRP tumors and limitations of HRD detection. Addressing this need is critical to reduce toxicity and improve disease management.

## 1. Introduction

Epithelial ovarian cancer (EOC) is the deadliest gynecologic malignancy and the fifth in cancer deaths among women in the United States [1]. EOC exhibits the lowest 5-year survival rate among gynecologic malignancies. The primary reason for this unfavorable prognosis is that the majority of cases are diagnosed with advanced disease, with 75–80% identified at clinical stages III or IV [2]. A prospective UK study showed that 5-year survival was 87% for women diagnosed at stage I, 62% for stage II, 26% for stage III, and 14% for stage IV [3]. Germline mutations in the susceptibility genes, *BRCA1* and *BRCA2*, also affect survival. It has been shown that 5-year overall survival for non-carriers is 36% (95% confidence interval (CI) 34–38) compared to 44% (95% CI 40–48) for *BRCA1* carriers and 52% (95% CI 46–58) for *BRCA2* carriers [4]. However, these data are prior to the poly ADP-ribose polymerase inhibitors (PARPis) era, which may favorably affect clinical outcomes in this group of patients. The current standard of care for patients with EOC is evolving. However, depending on the stage and molecular characteristics, it should include complete cancer resection, platinum/taxane chemotherapy, and antiangiogenics and/or PARPi inhibitors.

EOC has six main histotypes: (1) high-grade serous (HGSOC) cases that account for up to 70% of all cases, (2) endometrioid (ENOC) (~10%), clear cell (CCOC) (~10%), mucinous (MOC) (~3%), low-grade serous carcinomas (LGSOCs) (<5%), and carcinosarcoma which is a mixed epithelial and mesenchymal malignancy (1–4%) [5,6]. HGSOC is the most frequent histotype, and these tumors are characterized by *TP53* mutations [7], *BRCA* pathway alterations [8], and defects in homologous recombination (HR) DNA repair [9]. HGSOC tumors are also characterized by genomic instability and widespread copy number changes [9].

In the context of ovarian cancer susceptibility, having a family history of breast or ovarian cancer remains the most significant risk factor for EOC. Among genetic risk factors, germline mutations in the high-penetrance susceptibility genes *BRCA1* and *BRCA2* confer lifetime EOC risks of approximately 44% and 17% by age 80, respectively [10]. These genes are pivotal to the homologous recombination repair (HRR) pathway, and defects in DNA repair mechanisms are often due to pathogenic mutations in homologous recombination deficiency (HRD)-related genes such as *BRCA1* and *BRCA2*, or to epigenetic silencing through promoter methylation [11].

Due to the treatment advances of PARPis, HRD tumors have been extensively explored over the last two decades. The main principle involved in this therapy is the synthetic lethality mechanism, where the inhibition of PARP (enzymes involved in DNA repair), combined with the inability to repair double-strand breaks in HRD tumors, results in the accumulation of DNA damage and ultimately leads to cell death [12].

**Figure 1:**
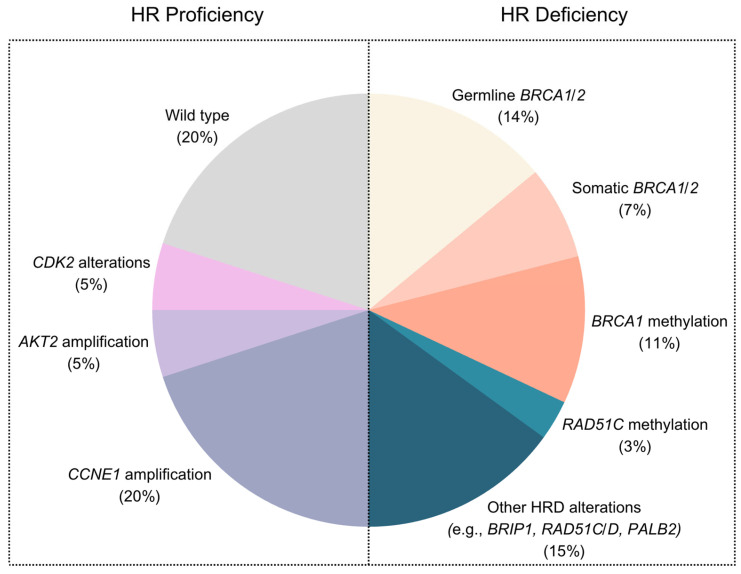
Mutational landscape of HGSOC carcinomas divided by HRD and HRP tumors.

Approximately 50% of all HGSOC cases have tumors with HRD [9] (Figure 1). However, much less is known about the other half of the cases that do not have defects in the homologous recombination pathway, often referred to as homologous-recombination-proficient (HRP) tumors. HGSOC patients with HRP tumors have a poor prognosis due to treatment resistance to both primary and targeted treatments, as well as shorter survival times [13]. Therefore, focusing on HGSOC patients with HRP tumors to better understand their cancer biology and develop new treatment options is of great clinical interest.

## 2. Homologous Recombination Deficient Tumors

Germline mutations in *BRCA1* and *BRCA2* are well-established contributors to HRD, but this perspective is somewhat simplistic, as HRD arises from a broader range of genetic and epigenetic mechanisms [5,8,11,13]. Mutations affecting other HRR genes, including *ATM*, *PALB2*, *RAD51*, and *CHEK2*, disrupt different stages of the HRR pathway and have also been identified as significant drivers of HRD [9,11,13,14]. These mutations emphasize the complexity of the DNA repair pathway and their critical role in maintaining genomic stability. In addition, epigenetic changes, particularly methylation of the *BRCA1* promoter, contribute to HRD by silencing gene expression and causing functional deficiencies without underlying genetic mutations [9,11,13,14]. Loss of heterozygosity (LOH), characterized by the loss of the wild-type allele, further exacerbates defects associated with mutations or epigenetic silencing of HRR genes and is considered another hallmark of HRD [13,14]. Chromosomal rearrangements and large deletions are structural variations that also significantly impair HRR function, contributing to the HRD phenotype [5,9,11,13,14]. HRD can result not only from germline but also from somatic mutations in HRR genes [9,11,13,14]. These somatic alterations expand the clinical relevance of HRD by affecting patients without inherited cancer predisposition [9,11,13,14]. The complexity of HRD mechanisms highlights the need to look beyond *BRCA1/2* mutations to include a broader range of genetic, epigenetic, and structural variations, making a comprehensive understanding essential for accurate HRD assessment and the optimization of therapeutic strategies such as PARP inhibitors and other precision oncology approaches in HRD tumors [9,13,14].

## 3. Homologous-Recombination-Proficient Tumors

HRP ovarian carcinomas are likely to be a heterogeneous group of diseases, and genetic alterations in signaling pathways that regulate the cell cycle are a common feature (Figure 1) [15,16].

Approximately 20% of the HGSOC tumors present *CCNE1* amplification or gain (Figure 1) [15]. AKT2 amplification (~5%) and CDK2 alterations (~5%) are also commonly associated with HRP tumors [17,18,19,20]. The tumor suppressor genes *RB1* and *NF1*, which act as a negative regulator of the RAS pathway, are also lost due to mutations and gene breakage [15]. Additionally, a small proportion of HGSOCs is deficient in mismatch repair (MMR), which might be commonly accepted as an HRP feature.

More recently, it has been shown that fold-back inversions are another potentially prevalent molecular characteristic in HRP tumors, and tumors with this structural variation are associated with inferior survival [21].

Whole-genome doubling, a hallmark of genomic instability, has been increasingly recognized in homologous-recombination-proficient tumors. This phenomenon may contribute to tumor heterogeneity and therapeutic resistance, emphasizing the need to explore its clinical and prognostic significance further [22].

Focusing on HGSOC patients with HRP tumors is extremely important and an enormous clinical need to avoid unnecessary toxicity and increase disease control. In the PAOLA trial, patients received olaparib plus bevacizumab as maintenance treatment following a response to first-line platinum chemotherapy [23]. The groups with HRD and *BRCA1/2*-mutated tumors demonstrated improved progression-free survival (PFS) in the PARPi group compared to the placebo. However, the cohort of patients with HRD-negative tumors did not show any benefit, achieving a PFS of 16.9 months with PARPis plus bevacizumab compared to 16.0 months with placebo (hazard ratio for disease progression or death: 0.92; 95% confidence interval: 0.72 to 1.17) [23]. In the PRIMA trial, patients with stage III HGSOC with residual disease and stage IV HGSOC were randomized to receive niraparib or placebo as maintenance treatment [24]. This study demonstrated, for the first time, that patients with HRP tumors experienced a benefit in PFS with the PARPi treatment (PFS of 8.1 months versus 5.4 months; HR 0.68; CI 0.49–0.94; *p* = 0.02) [24]. However, the final results showed no benefit in OS for the HRP population [24].

In the era of precision medicine, selecting the appropriate patients likely to benefit from the targeted therapy is crucial. Therefore, new targeted treatments must be identified for HGSOC patients with HRP tumors and those with HRD tumors who have acquired HR proficiency. This review examines the current understanding of HGSOC patients with HRP tumors and provides insights into potential new targets for novel therapies.

## 4. CCNE1 Amplification

*CCNE1* amplification/gain and/or overexpression has been considered a prevalent molecular feature in HGSOC patients with HRP tumors. It occurs in approximately 20% of HGSOC cases, has been associated with a poor outcome, might be predictive of primary treatment response, and might be targetable [9,15]. *CCNE1* forms a complex with *CDK2* by phosphorylation of downstream targets, such as the tumor suppressor *RB1*, and it regulates the G1–S transition and has kinase-independent functions, including in DNA replication [25]. A hyper-proliferative phenotype increases the speed at which cells progress through the G1/S-phase restriction point, leading to replicative stress, whole genome duplication, and additional gene dysregulation that are responsible for proliferation and cell survival [26,27].

*CCNE1* amplification and/or overexpression is largely mutually exclusive with germline *BRCA1* and *BRCA2* mutations [9,11,28] and has been reported as the dominant molecular alteration associated with primary treatment failure [29], although some studies showed controversial data [14]. Patients with tumors with *CCNE1* amplification usually have poor response to platinum-based chemotherapy and limited response to PARPis [8,11,14]. Whole-genome sequencing data showed the overall survival difference between patients with HRD tumors, *CCNE1*-amplified tumors, and those with none of these features (HR/*CCNE1* negative) [9]. Two datasets were analyzed: an in-house cohort of 80 patients and a validation TCGA cohort of 279 patients. In both analyses, patients with HRD tumors had significantly better overall survival than *CCNE1*-amplified tumors or HR/*CCNE1*-negative tumors [9]. More recently, *CCNE1* gain was assessed among HR wild-type tumors [14]. *CCNE1*-amplified tumors had shorter survival time and significantly poorer survival compared to non-*CCNE1*-amplified tumors [14]. In addition, *CCNE1*-amplified tumors also had the lowest levels of infiltrating immune cells compared to HRD tumors, which suggest that these patients might also not benefit from immune-checkpoint inhibitors [14].

Identifying other biological candidates for targeting *CCNE1* amplified tumors can be challenging due to the low frequency of other molecular events in this group of tumors, such as *BRCA1/2* wild-type and low immune cell infiltration. The National Comprehensive Cancer Network (NCCN) guidelines currently recommend testing for germline and somatic *BRCA1/2*, other HR pathway genes, and microsatellite instability or DNA mismatch repair, which would fail to identify tumors with *CCNE1* alterations (NCCN Guidelines Version 1.2022—Epithelial Ovarian Cancer/Fallopian Tube Cancer/Primary Peritoneal Cancer). There are several emerging targeted therapies in clinical trials targeting *CCNE1*-amplified tumors. The first attractive target for treating patients with these tumors was *CDK2*, which plays an essential role in activating the *CDK2*/cyclin E1 complex. *CDK2* inhibitors showed promising results in in vitro studies [17,28], but none of the agents used in clinical trials passed phase II. At least seven trials were performed with unsuccessful results: (1) AT7519 (AT7519M, Astex Therapeutics Ltd., Cambridge, UK); (2) AG-024322 (Pfizer, New York City, NY, USA); (3) Dinaciclib (MK7965, SCH727965, Merck & Co., Rahway, NJ, USA); (4) CYC065 (Cyclacel Pharmaceuticals, Berkeley Heights, NJ, USA); (5) Ronaciclib (BAY 1000394, Bayer, Leverkusen, Germany); (6) TG02 (Tragara Pharmaceuticals, San Diego, CA, USA); (7) Milciclib (PHA 848125, Tiziana Life Sciences, London, UK) (Table 1).

Co-amplification patterns could also be of interest for clinical applications [18]. *AKT2* amplification has been shown to have poor prognosis in HGSOC and has been associated with *CCNE1* amplification [18,19,20]. This co-amplification could be explained by the proximity of chromosome 19q [18]. A few clinical trials have investigated the activity of drugs targeting AKT both as monotherapy and in combination with other targeted therapies [30,31]. However, further studies are still needed [30,31].

A promising class of drugs to treat patients with CCNE1-amplified tumors is the Wee1 inhibitors. Adavosertib, a Wee1 inhibitor, plus gemcitabine, was evaluated in platinum-resistant or platinum-refractory recurrent advanced ovarian cancer [32] (NCT02151292, Table 1). This trial showed significantly extended progression-free and overall survival by adding adavosertib to gemcitabine in platinum-resistant or platinum-refractory advanced HGSOC. However, more extensive confirmatory studies are still required [32]. In addition, the IGNITE trial is a signal-seeking trial of adavosertib targeting recurrent HGSOC with CCNE1 overexpression with and without gene amplification [33] (Table 1). In the cohort of tumors that were overexpressed but not amplified, an overall response rate (ORR) of 53% was achieved [33]. Adavosertib was also evaluated with or without PARPi (olaparib) in treating recurrent ovarian cancer patients in the EFFORT trial [34] (Table 1). That study indicated that the Wee1 inhibitor, when used alone or with olaparib, showed effectiveness in patients with PARP inhibitor-resistant ovarian cancer [34]. While they observed some severe side effects in both groups, these were typically manageable with support, occasional pauses in treatment, and dose reductions as necessary [34]. Further analyses are underway to determine which patients benefited the most.

## 5. Replication Stress

Tumors enriched for biomarkers of replicative stress, such as CCNE1 amplification, are characteristic of HRP tumors and have also been suggested to be more likely to respond to ATR inhibitors [35], even though correlative work is still needed to confirm this hypothesis. Based on the prevalence of DNA replication stress in HGSOC tumors, inhibition of ATR has been recently examined as a multicenter, open-label, randomized, phase II trial (NCT02595892) (Table 1) [35]. It was shown that progression-free survival was increased in platinum-resistant HGSOC cases when adding the ATR inhibitor berzosertib to gemcitabine compared to gemcitabine alone [35]. Although the study did not conduct subgroup analysis, this strategy could represent a promising option for platinum-resistant disease, including the proficient subgroup [35]. The same ATR inhibitor, M6620, was tested in a phase I trial as monotherapy or combined with carboplatin [36]. It was found that this drug was well tolerated, had target engagement, and a preliminary antitumor response was observed (Table 1) [36]. Other ongoing trials are testing AZD6738 alone and in combination with olaparib (NCT03682289) as well as BAY 1895344 in combination with chemotherapy (NCT04491942) (Table 1). Both trials are still recruiting patients; their results should be published in the next few years.

## 6. High Fold-Back Inversions

Fold-back inversions (FBIs) represent a relatively rare but potentially crucial structural variation in HRP tumors. Mechanistically, FBIs in ovarian cancer tumors may be due to rearrangements during the breakage–fusion–bridge cycles [37]. An FBI occurs when the bridge breaks and reattaches in the opposite direction, reversing a chromosome segment. Therefore, this structural variation consists of two copies of a duplicated genome region facing opposite directions from the breakpoint [38].

Wang et al. analyzed whole-genome point mutation and structural variation patterns of 59 HGSOCs and identified a high prevalence of FBIs associated with inferior survival [21]. Their results were replicated in two independent cohorts (*n* = 576 cases). They showed that tumors with high FBIs had a worse prognosis than those with low FBIs. In addition, they also showed that FBIs were associated with poor prognostic features such as *CCNE1* amplification, characteristic of HRP tumors [21]. Therefore, high vs. low FBI rates could be considered a prognostic and predictive biomarker for treatment with PARP inhibitors [38]. Wang and colleagues suggested that FBI profiles could stratify patients with HGSOC tumors. Most high-FBI tumors exhibit *BRCA1/2* wild-type status and *CCNE1* amplification, with low *MECOM/MYC* amplification and *PTEN* deletion. In contrast, HRD tumors show higher *MECOM/MYC* amplification and *RB1* loss [21]. In light of these data, high versus low FBI rates could be evaluated as potential prognostic and predictive biomarkers for treatment with PARP inhibitors, warranting further investigation.

Unfortunately, new therapies targeting fold-back inversions remain unknown (Table 1). Treating tumors with these structural variations can be challenging because they often exhibit complex genomic alterations. Nevertheless, it is essential to identify harbor activating mutations in oncogenes that can be targeted or identify specific biological pathways that are dysregulated in these tumors.

## 7. Antibody Drug Conjugates (ADCs) Targeting Folate Receptor-Alpha

An antibody-drug conjugate (ADC) consists of a monoclonal antibody that is chemically bonded to a cytotoxic drug through a linker [39]. This design allows for the precise targeting of cancer cells while delivering a highly potent therapeutic agent, leading to efficient and selective destruction of tumor cells with a manageable toxicity profile [39].

It has been known for years that increased folate receptor-alpha expression is characteristic of EOC, particularly in HGSOC, as opposed to normal adult tissues, which typically show limited folate receptor-alpha expression [40,41]. The folate receptor-alpha inhibitors have been tested in platinum-resistant cases. When resistance occurs, many HRD tumors eventually become HRP after undergoing platinum-based or PARPi treatments. This makes this emerging therapy potentially interesting for HRP tumors, whether they are primary or have acquired proficiency.

Combined with chemotherapy, a clinical trial using a folate receptor-alpha inhibitor named vintafolide showed improved progression-free survival in patients with platinum-resistant ovarian cancer [42]. However, the following phase III trial did not significantly improve overall survival when using this regimen [43]. More recently, a global, single-arm study named SORAYA evaluated the use of mirvetuximab soravtansine (MIRV) in platinum-resistant HGSOC patients [41] (Table 1). MIRV is composed of an antibody anti-folate receptor-alpha, a cleavable linker, and maytansinoid DM4, a potent tubulin-targeting agent. The trial focused on patients with resistant tumors that overexpressed folate receptor-alpha, and they demonstrated that MIRV had a consistent clinically meaningful antitumor activity and favorable tolerability and safety in the targeted population [41]. These data were further confirmed in the randomized phase III trial MIRASOL, where MIRV demonstrated a benefit in overall survival (OS) compared to chemotherapy in the setting of platinum-resistant disease [44] (NCT04209855) (Table 1). This is the first ADC to receive FDA approval in ovarian cancer [45]. Furthermore, the phase III GLORIOSA trial is ongoing to assess the efficacy of MIRV in combination with bevacizumab maintenance therapy compared to bevacizumab alone, following platinum-based chemotherapy in patients with folate receptor-alpha high-grade platinum-sensitive disease [46]. If positive, this strategy could become a maintenance treatment option for patients with HRP.

## 8. ADCs Targeting NaPi2B

The sodium-dependent phosphate transport protein 2B (NaPi2B) plays a key role in maintaining phosphate homeostasis, and its dysfunction may contribute to the development of certain pathologies, including hyperphosphatemia. Elevated inorganic phosphate concentrations within the tumor microenvironment, compared with normal tissues, have been identified as a potential marker of tumor progression [47]. NaPi2B is expressed in approximately 66% to 90% of HGSOCs [45,47]. Lifastuzumab vedotin (LIFA) is an ADC consisting of a humanized anti-NaPi2b monoclonal antibody linked to the potent antimitotic agent monomethyl auristatin E. Its efficacy was evaluated in a phase II trial in the platinum-resistant setting [48]. Although the ORR was 34%, no statistically significant improvement in PFS was observed [48]. TUB-040 is a highly homogeneous, hydrophilic ADC targeting NaPi2B, conjugated to the cytotoxic payload exatecan, a potent topoisomerase I inhibitor with a robust bystander effect [49]. The NAPISTAR 1–01 trial is an ongoing phase I/IIa study evaluating TUB-040 in patients with platinum-resistant ovarian cancer (NCT06303505) [49] (Table 1). The efficacy of another NaPi2B-targeting ADC, upifitamab rilsodotin (UpRi), was assessed in the phase II UPLIFT trial [50] (Table 1). The drug demonstrated an ORR of 34% in a population of NaPi2B-high patients with platinum-resistant ovarian cancer [45,50]. Currently, UpRi is being tested as maintenance treatment in the platinum-sensitive population in the phase III UP-NEXT trial (Table 1). While NaPi2B has been identified as a promising biomarker candidate for the platinum-resistant ovarian cancer population, including patients with HRP disease, its clinical utility remains to be established. Confirmation will require results from larger, well-designed clinical trials to validate its prognostic and predictive value before it can be incorporated into routine practice.

## 9. ADCs Targeting HER-2

Human epidermal growth factor receptor 2 (HER2) is a transmembrane tyrosine kinase receptor that plays a key role in promoting cell proliferation, differentiation, and survival, and HER2 expression can occur in a wide range of solid tumors [51]. Trastuzumab deruxtecan (T-DXd) is an ADC composed of an anti-HER2 monoclonal antibody, a cleavable linker, and a topoisomerase I inhibitor payload [51]. Its clinical application in HER2-positive breast cancer is already well established, and more recently, its agnostic application was studied in the Destiny-PanTumor02 trial, which included three cohorts of gynecological tumors [51]. Regarding the ovarian cancer cohort, patients with HER2 immunohistochemistry expression were included. The median previous lines of treatment were three [51]. T-DxD demonstrated an ORR of 45% in the entire cohort and 63.6% in patients with HER2 3+ expression [51].

## 10. Mismatch Repair Deficiency

A small proportion of HGSOC cases might have germline mutations in MMR genes, which would explain the rationale for exploring immunotherapy [52]. However, immune checkpoint inhibitors have been showing very limited benefit in ovarian cancer [53].

MMR deficiency (MMRd) typically arises from mutations in genes encoding MMR proteins such as MLH1, MSH2, MSH6, and PMS2. These proteins are responsible for identifying and correcting mismatches in nucleotides or through the methylation of the MLH1 gene promoter. These MMR errors result in microsatellite instability (MSI) and short repetitive sequences in DNA. Tumors characterized by a high degree of MSI (MSI-H) exhibit a significantly increased number of somatic mutations, leading to the expression of many neoantigens and making them potentially more responsive to immunotherapy when compared to tumors with fewer mutations [54]. It has been reported that MSI-H or MMRd features are present in approximately 30% of endometrial cancers, 20% of colon or gastric cancers, and less than 5% for most other tumor types [54].

Immune checkpoint inhibitors have demonstrated significant efficacy in treating solid tumors with MMR deficiency. In ovarian cancer, they could represent a therapeutic option for MMRd tumors that are refractory to standard treatment [55]. However, only a small proportion of HGSOCs have been identified as MMRd. In the KEYNOTE-158 trial, a phase II study evaluating pembrolizumab in MMRd cancers, the ovarian cancer cohort included 15 patients and achieved an ORR of 33.3% [55]. The use of Nivolumab and pembrolizumab in MMRd tumors, regardless of histology and cancer site, is currently approved by The Food and Drug Administration (FDA). However, there is a limited availability of large datasets on MMR deficiency prevalence in HGSOC. A systematic review and meta-analysis identified that a notable minority of ovarian cancers are MMR deficient [52]. They observed MMR deficiency in all histotypes, but as expected, it was most commonly found in endometrioid tumors [52]. This study did not present any data on HR status [52].

Immune checkpoint inhibitor trials have yielded dismal results regarding its effectiveness in HGSOC [56,57,58,59,60]. Most of these studies did not specifically focus on MMR-deficient tumors, which could explain why they could not establish treatment effectiveness. Understanding which tumors are more likely to respond to immunotherapies is fundamental. Most of the studies have described that HRD tumors are associated with higher neoantigen loads, elevated tumor-infiltrating lymphocytes, and increased expression of immune pathway genes, and this could make them more receptive to immunotherapy [61,62]. Treating HGSOC patients with HRP tumors is further complicated by the presence of an immunosuppressive tumor microenvironment (TME) [63]. Therefore, immunotherapy is thought to be less effective in this group of patients.

## 11. Other Molecular Alterations and New Emerging Therapies

Finding better alternatives for patients with HRP tumors has been challenging because previous well-known analyses of independent TCGA datasets did not find any significant type or frequency of driver mutations in this group of tumors or no patterns of methylation or miRNA expression [9,11]. A univariate survival analysis was performed by comparing *BRCA1/2* mutated cases, wild-type cases, and epigenetically silenced *BRCA1* cases by the TCGA Network researchers [11]. Overall survival was better in the *BRCA1/2*-mutated cases, as expected, due to their defect in the HR pathway, leading to better response to primary treatment [11]. Interestingly, epigenetically silenced *BRCA1* cases had similar survival to *BRCA1/2* wild-type cases, suggesting that mutually exclusive genomic and epigenomic mechanisms inactivate *BRCA1*, and this inactivation mechanism influences patient survival [11]. The poorer overall survival is consistent with HRP tumors, but the mutation status of other HRD genes is unknown.

HRD tumors may also evolve into HRP via secondary genomic or epigenomic alterations that restore HRR activity due to selective pressure primarily from prior platinum and/or PARPi therapy [16]. HRP tumors can also be induced by other mechanisms of treatment resistance in HGSOC, such as the demethylation of *BRCA1* and *RAD51C*. Methylation is a chemical modification of DNA that can affect specific genes’ expression and function. Adding methyl groups to DNA is associated with gene silencing, while demethylation, which involves the removal of methyl groups, can reactivate genes. It is known that *BRCA1* and *RAD51C* can be epigenetically silenced through hypermethylation of their promoter regions in HGSOC. This silencing can lead to a loss of function in these genes and contribute to the development and progression of HGSOC due to defects in the HR pathway. PARP inhibitors target these types of defects in the HR pathway. Therefore, restoring these defects through demethylation of *BRCA1* and *RAD51C* promoter regions leads to resistance to this treatment. Targeting the demethylation of *BRCA1* and *RAD51C* promoter regions may be a potential strategy to overcome platinum-based and PARP inhibitor resistance. The use of the demethylating agents has already been tested in clinical trials (Table 1). A phase II trial tested decitabine in combination with carboplatin in patients with platinum-resistant ovarian cancer and found that adding the drug reduced rather than increased the efficacy of carboplatin. It was also found that the drug was complicated to deliver [64]. A pre-clinical study also showed that guadecitabine, a methyltransferase inhibitor, in combination with the PARPi talazoparib, inhibited breast and ovarian cancers harboring either wild-type- or mutant-*BRCA1/2* [65]. This combination of drugs still needs further clinical exploration.

More recently, *CARM1*, an arginine methyltransferase that is often overexpressed in human cancers, has been explored in HGSOC [66]. It has been shown that approximately 20% of HGSOC cases have *CARM1* amplification/overexpression. Importantly, HGSOC tumors with high expression of *CARM1* are typically HRP and mutually exclusive with *BRCA1* and *BRCA2* mutations [66]. Targeting HGSOC tumors with high expression of *CARM1* has been tested recently by using EZH2 inhibitors and has shown promise in pre-clinical studies [67]. EZH2 inhibition upregulates *MAD2L2*, which plays a role in choosing between homologous recombination and non-homologous end-joining (NHEJ)-mediated repair and sensitizes HRP tumors to PARPis [67]. *CARM1* promotes *MAD2L2* silencing, and *EZH2* inhibition upregulates *MAD2L2* to decrease DNA end resection. NHEJ and chromosomal abnormalities are increased, causing a mitotic catastrophe in PARP inhibitor-treated HRP cells [67]. It has been shown that the EZH2 inhibitor sensitizes *CARM1*-high to PARP inhibitors in both orthotopic and patient-derived xenografts [67]. Interestingly, it has been shown that cells that overexpress *CARM1* also overexpress *CCNE1* [68]. *CARM1* was shown to act as a coactivator for *CCNE1*, suggesting that *CARM1* and *CCNE1* overexpression might be associated [68].

Studies using epidermal growth factor receptor (EGFR) inhibitors have presented controversial results. While some studies have shown improved response rates and progression-free survival when using EGFR inhibitors alone or in combination with chemotherapy, others found results that suggested that these drugs may worsen outcomes in specific patient populations. Other therapies such as phosphoinositide 3-kinase (PI3K) [69], Bromodomain and extra-terminal (BET) [70], and Notch inhibitors [71] showed promising results in pre-clinical studies; however, no significant benefit was observed in clinical trials.

It was also shown that the histone deacetylase inhibitor (HDACi) entinostat enhanced the effect of olaparib in reducing cell viability and clonogenicity in HR-proficient ovarian cancer cells [72]. Despite this, in a phase II trial, it failed to demonstrate activity in platinum-resistant ovarian cancer when combined with immunotherapy [73].

Lastly, exploratory analysis showed that HIPEC may benefit HRD patients. In the OVHIPEC trial, a stratified analysis revealed that HIPEC provided the greatest benefit in HRD/*BRCA*-wt tumors (HR 0.44; 99% CI 0.21–0.91) compared to non-HRD/*BRCA*-wt (HR 0.82; 99% CI 0.48–1.42) and *BRCA1/2* tumors, where no benefit was observed (HR 1.25; 99% CI 0.48–3.29) [74]. These findings suggest that patients with HRD tumors lacking *BRCA1/2* pathogenic mutations derive the most significant advantage from HIPEC treatment [74].

## 12. Additional Considerations Regarding Homologous Recombination Status in HGSOC

Patients with HRP tumors face many more challenges that need to be addressed. One such challenge is that determining HR deficiency or proficiency can be complex [75], with barriers including financial constraints, limited accessibility, logistical issues, and psychological concerns. Current HR status detection methods, such as myChoice CDx and FoundationOne CDx, face limitations and lack standardization due to different definitions of HR deficiency. The use of binary cut-off to continuous measures in these clinically validated tests introduces significant limitations, reducing statistical power and potentially underestimating the results [76]. In addition, these tests can be costly, have availability constraints, and require substantial high-quality cancer tissue, making interpretation challenging. Alternative approaches, like base-substitution signature 3 [77], HRDetect [78], and copy number signatures [79], aim to overcome these challenges by distinguishing HR deficiency from proficiency based on genomic signatures and chromosome instability types. Importantly, these alternative approaches are currently only used in research settings and, as such, have not been clinically validated.

## 13. Discussion

Ovarian cancer treatment has faced significant advances in recent years by exploring HRD and the consequent genomic instability it causes. However, around 50% of the HGSOC patients are HRP, and further research is needed to understand the underlying mechanisms that drive carcinogenesis and develop personalized treatment strategies for patients with HRP tumors. Identifying key driver mutations and pathway alterations in HRP tumors would be incredibly helpful in discovering new targeted treatments. Substantial advances have been made in the past few years concerning targeted treatment options for HGSOC patients, such as PARPis, but these new therapies are not focused on patients with HRP tumors. Other targeted therapies have been studied in HGSOC patients but have yet to be analyzed regarding HR status.

Immune checkpoint inhibitors, for instance, were expected to show promise in treating ovarian cancer, particularly in patients with tumors exhibiting MMR deficiency and/or high levels of immune cell infiltration, but results have been disappointing [57,58,59,80]. In addition, MMR deficiency prevalence in HGSOC is very low, and no available data indicate whether MMR deficiency could be more common in HRP compared to HRD tumors. Furthermore, HRD tumors have been found to exhibit higher neoantigen loads, increased tumor-infiltrating lymphocytes, and elevated expression of immune pathway genes when compared to HRP tumors. These observations, combined with the fact that HRP tumors also present an immunosuppressive TME, once again suggest that immunotherapy is less likely to be effective in patients with HRP tumors, demonstrating the importance of further exploring various features in these tumors [63].

Throughout this review, we highlighted numerous HGSOC molecular features directly or indirectly associated with HRP tumors, many of which also present potential new emerging therapies that could target this group of neglected patients. *CCNE1* amplification occurs in 20% of HGSOC cases and is largely mutually exclusive with *BRCA1/2* mutations. Although no benefit was observed with CDK inhibitors, Wee1 inhibitors are still undergoing testing and are currently demonstrating promising results in clinical trials. Demethylation of *BRCA1* and *RAD51C* is also mutually exclusive with *BRCA1/2* mutations, and demethylation agents, such as DNA methyltransferase inhibitors, in combination with platinum-based therapies or PARPis, are currently being investigated. Once again, *CARM1* amplification/overexpression is mutually exclusive with *BRCA1/2* mutations. This feature can be found in approximately 20% of HGSOC patients. The EZH2 inhibitor has sensitized tumors with CARM-1 amplification/overexpression to PARP inhibitors in orthotopic and patient-derived xenografts. There is an urgent need for additional and more extensive research specifically focused on HGSOC patients with HRP tumors. New molecular features can be discovered only by devoting appropriate attention to this issue.

We also explored the role of ADCs targeting additional molecular features in HGSOC associated with HRP tumors. High folate receptor-alpha expression has been associated with platinum and PARPi resistance. In cases where resistance develops, numerous HRD tumors may ultimately regain HR proficiency following platinum-based or PARPi treatments. Folate receptor-alpha inhibitors have demonstrated promising results in recent clinical trials. NaPi2B and HER2 expression were also explored in this review in the context of ADCs.

In addition, acquired treatment resistance in HGSOC is a common challenge in managing this disease. Resistance mechanisms involve the restoration of HR proficiency, intratumoral heterogeneity, and BRCA-independent pathways, leading to a lack of effective treatment options for patients with resistant tumors. Due to individual variations in resistance, tailored treatment plans are essential, and ongoing research aims to uncover resistance mechanisms for the development of more effective therapies. Encouraging clinical trial participation for platinum-resistant patients has shown improved survival and should be prioritized.

The importance of better understanding HRP tumors goes beyond HGSOC and even beyond ovarian tumors. The non-HGSOC histotypes (low-grade serous, endometrioid, clear cell, mucinous, and carcinosarcomas), for instance, can be classified as HRP tumors. Therefore, tailored treatments targeting these tumors with unique underlying biology are also needed. In addition, the prevalence of HRP tumors in various cancers poses a challenge. An analysis of pan-cancer cohorts recently showed data in other tumors where *BRCA1/2* mutation is frequent. In prostate and pancreatic cancers, for instance, 90–95% of primary and 85% of metastatic tumors were classified as HRP, emphasizing the need for further research on HRP tumors across different cancer types [81].

## 14. Conclusions

HRP is a large, heterogeneous subset with distinct molecular drivers that are potential therapeutic targets. Understanding cancer biology and developing new treatment options for this group of neglected ovarian cancer patients is a critical clinical need to avoid unnecessary toxicity and improve ovarian cancer disease management.

## Figures and Tables

**Table 1:** Summary of HRP tumor’s molecular features and its potential new targeted treatments.

Molecular Alteration	Main Features	Therapies/ Potential Therapies	Clinical Trials	Trial Status
*CCNE1* amplification/overexpression	Occurs in 20% of HGSOC cases Largely mutually exclusive with *BRCA1/2* mutations	CDK inhibitors with CDK2-specific activity	AT7519 (AT7519M, Astex Therapeutics Ltd., Cambridge, UK) AG-024322 (Pfizer) Dinaciclib (MK7965, SCH727965, Merck & Co.) CYC065 (Cyclacel Pharmaceuticals) Ronaciclib (BAY 1000394, Bayer) TG02 (Tragara Pharmaceuticals) Milciclib (PHA 848125, Tiziana Life Sciences)	None have passed phase II
		Wee1 inhibitors	NCT02151292 (NCI): adavosertib plus gemcitabine	Active, not recruiting: reported benefit in PFS
			IGNITE (AstraZeneca, Cambridge, UK): adavosertib	Completed, ORR 53%
			NCT02272790 EFFORT (AstraZeneca): adavosertib with or without olaparib	Completed
High prevalence of replication stress	Most of the HGSOC cases, both HRD and HRP tumors In HRP, tumors are caused by premature entry into the S phase due to *CCNE1* amplification.	ATR inhibitors	NCT02157792: Phase I trial of M6620 (VX-970) as monotherapy or in combination with carboplatin (Vertex Pharmaceuticals, Boston, MA, USA)	M6620 was well tolerated, with target engagement and preliminary antitumor responses observed
			NCT02595892: Phase II trial testing gemcitabine hydrochloride alone or with M6620—National Cancer Institute (NCI)	Active, not recruiting
			NCT03682289: Phase II trial testing AZD6738 alone and in combination with olaparib—AstraZeneca.	Recruiting
			Phase I trial testing BAY 1895344 in combination with chemotherapy—NCI	Recruiting
			NCT02595892: Phase II trial testing berzosertib plus gemcitabine versus gemcitabine alone in platinum-resistant HGSOC	Completed; benefit in PFS for the interventional arm
Fold-back inversions (FBI)	Most tumors with high FBI have *BRCA1/2* wild-type status Associated with poor prognostic features such as *CCNE1* amplification	NA	NA	NA
High folate receptor alpha expression	Associated with platinum and PARPi resistance	Mirvetuximab soravtansine	NCT04296890: SORAYA (ImmunoGen, Inc., Waltham, MA, USA): MIRV	Completed: meaningful antitumor activity
			NCT04209855: MIRASOL (ImmunoGen, Inc.): MIRV	Completed
			NCT05445778: GLORIOSA (Immunogen, Inc.)	Recruiting
Sodium-dependent phosphate transport protein 2B (NaPi2B)	Associated with platinum resistance	ADC Upifitamab rilsodotin (UpRi)	NCT03319628: UPLIFT	Active, not recruiting
			NCT05329545: UP-NEXT	Terminated by sponsor; waiting results
		ADC TUB-40	NCT06303505	Recruiting
MMR deficiency	High degree of microsatellite instability Large number of neoantigens	Checkpoint inhibitors	No trials have been conducted in the context of ovarian cancer HRP tumors	NA
Demethylation of *BRCA1* and *RAD51C*	Mutually exclusive with *BRCA1/2* mutations Demethylation of a single copy of initially *BRCA1* and *RAD51C* methylated genes will restore the protein due to treatment pressure	Demethylating agents in combination with platinum-based therapies or PARPis	Phase II trial: Decitabine in combination with carboplatin vs. carboplatin alone (Cancer Research UK. CRUKD/07/065 and Eisai, Tokyo, Japan)	Terminated (no efficacy observed)

## Abbreviations

ADC	Antibody drug conjugate
BET	Bromodomain and extra-terminal
CCOC	Clear cell ovarian cancer
CI	Confidence interval
EGFR	Epidermal growth factor receptor
ENOC	Endometrioid ovarian cancer
EOC	Epithelial ovarian cancer
FBI	Fold-back inversions
FDA	Food and Drug Administration
HDACi	Histone deacetylase inhibitor
HER2	Human epidermal growth factor receptor 2
HR	Homologous recombination
HRD	Homologous recombination deficient
HRP	Homologous recombination proficient
HRR	Homologous recombination repair
HGSOC	High-grade serous ovarian cancer
LGSOC	Low-grade serous ovarian cancer
LOH	Loss of heterozygosity
MIRV	Mirvetuximab soravtansine
MMR	Mismatch repair
MOC	Mucinous ovarian cancer
MSI	Microsatellite instability
NaPi2B	Sodium-dependent phosphate transport protein 2B
NCCN	The National Comprehensive Cancer Network
NCI	National Cancer Institute
NHEJ	Non-homologous end-joining
ORR	Overall response rate
OS	Overall survival
PARPi	Poly ADP-ribose polymerase inhibitors
PFS	Progression-free survival
PI3K	Phosphoinositide 3-kinase
T-DXd	Trastuzumab deruxtecan
TME	Tumor microenvironment
UpRi	Upifitamab rilsodotin
